# Rapid Characterization of Fatty Acids in Oleaginous Microalgae by Near-Infrared Spectroscopy

**DOI:** 10.3390/ijms16047045

**Published:** 2015-03-27

**Authors:** Bin Liu, Jin Liu, Tianpeng Chen, Bo Yang, Yue Jiang, Dong Wei, Feng Chen

**Affiliations:** 1 School of Light Industry and Food Sciences, South China University of Technology, Guangzhou 510640, China; E-Mails: caisanrenju@163.com (B.L.); ly_mikeyang@163.com (B.Y.); fewd304@scut.edu.cn (D.W.); 2 Institute for Food and Bioresource Engineering, College of Engineering, Peking University, Beijing 100871, China; E-Mails: jliu@umces.edu (J.L.); tianpeng_chen@163.com (T.C.); 3 Institute of Marine and Environmental Technology, University of Maryland Center for Environmental Science, Baltimore, MD 21202, USA; 4 The School of Food Science and Technology, Jiangnan University, Wuxi 214122, China; E-Mail: jiangyue@tust.edu.cn; 5 Singapore-Peking University Research Centre for a Sustainable Low-Carbon Future, CREATE Tower 138602, Singapore

**Keywords:** biodiesel, *Chlorella*, fatty acids, microalgae, near-infrared spectroscopy

## Abstract

The key properties of microalgal biodiesel are largely determined by the composition of its fatty acid methyl esters (FAMEs). The gas chromatography (GC) based techniques for fatty acid analysis involve energy-intensive and time-consuming procedures and thus are less suitable for high-throughput screening applications. In the present study, a novel quantification method for microalgal fatty acids was established based on the near-infrared spectroscopy (NIRS) technique. The lyophilized cells of oleaginous *Chlorella* containing different contents of lipids were scanned by NIRS and their fatty acid profiles were determined by GC-MS. NIRS models were developed based on the chemometric correlation of the near-infrared spectra with fatty acid profiles in algal biomass. The optimized NIRS models showed excellent performances for predicting the contents of total fatty acids, C16:0, C18:0, C18:1 and C18:3, with the coefficient of determination (*R*^2^) being 0.998, 0.997, 0.989, 0.991 and 0.997, respectively. Taken together, the NIRS method established here bypasses the procedures of cell disruption, oil extraction and transesterification, is rapid, reliable, and of great potential for high-throughput applications, and will facilitate the screening of microalgal mutants and optimization of their growth conditions for biodiesel production.

## 1. Introduction

To date, fossil-derived fuels have still served as the main energy sources [1,2]. The ever-increasing energy demand, depleting reserves of fossil fuels, and environmental concerns, however, have urged the exploration of alternative energies that are green, renewable and sustainable [3]. Biodiesel, referring to a mixture of fatty acid methyl esters (FAMEs) produced by transesterification of oils, has attracted much attention due to its properties of being renewable, carbon neutral and portable for transporting use [4].

Microalgae are fast-growing photosynthetic organisms with the ability to accumulate high content of lipids, up to 70% of cell dry weight under certain growth conditions [5]. They have been considered better than oil crops for biodiesel production [1,6,7]. Among the oleaginous microalgae, *Chlorella* spp. are thought to be promising candidates of biodiesel feedstocks in that they are able to grow robustly for high cell density, produce high level of triacylglycerol, and serve as an ideal source for making biodiesel [8,9,10,11].

The key properties of biodiesel, such as cetane number, kinematic viscosity, oxidative stability, cloud point and cold filter plugging point, are largely determined by the composition of fatty acid methyl ester (FAME) [12,13,14,15,16]. Therefore, when evaluating the feasibility of biodiesel feedstocks, their fatty acid composition should be considered as an important indicator [10,17,18].

Gas chromatography-flame ionization detector (GC-FID) and Gas chromatography-mass spectrometry (GC-MS) represent the typical techniques to analyze the fatty acid profiles. Generally, these methods involve the energy-intensive and time-consuming procedures such as cell disruption, lipid extraction and transesterification and thus are less suitable for high-throughput screening applications [19,20]. Therefore, alternative techniques easier to conduct, but without significant loss of accuracy, are in sought for fatty acid analysis.

Near-infrared spectroscopy (NIRS) is such a technique; it is rapid, cost-effective, reliable, and of great potential for high-throughput applications. Fatty acids varying in chain length and unsaturation level possess different near-infrared spectra [21,22]. There have been several reports of employing NIRS for predicting individual fatty acids, such as C16:0, C18:0, C18:1 and C18:2, in pig adipose, lamb meat, chicken meat, milk powder and almond flour [23,24,25,26,27]. Recently, NIRS also demonstrated its applications in microalgae, but restricted to the quantification of lipid, carbohydrate, protein, and ash content [28,29,30,31,32,33]. The use of NIRS for individual fatty acid analysis in microalgae has not been reported, to the best of our knowledge. The aim of the present study was to establish a feasible NIRS method for the rapid analysis of microalgal fatty acid composition. With our optimized NIRS method, the microalgal fatty acid content and composition could be determined based on the NIR spectrum of a microalgal sample. Our work represents the first effort to develop a NIRS based method for the characterization of fatty acids in microalgae, which has great potential in high-throughput applications, in particular for the screening of microalgal mutants and optimization of their growth conditions for biodiesel production.

## 2. Results

### 2.1. Algal Samples and Near-Infrared (NIR) Spectra

**Figure 1:**
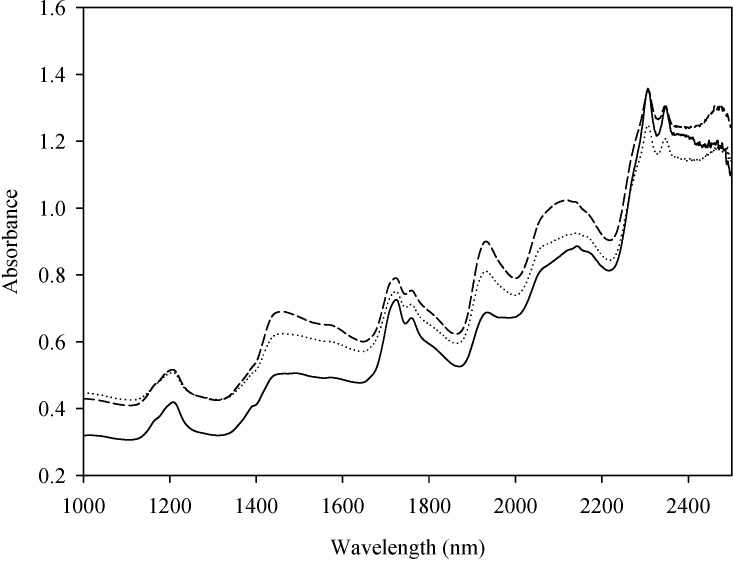
Average absorbance of *C. vulgaris* (long dash line), *C. protothecoides* (dotted line) and *C. zofingiensis* (solid line) samples over the range 1000–2500 nm.

All 159 samples were obtained by growing in the medium with a series of C/N ratios [34,35]. The average NIR spectra of 3 species of *Chlorella* were given in Figure 1 in the form of absorption spectra. The major NIRS absorption bands (Figure 1) of lipids were centered at 1195–1215 nm for CH_3_ and CH_2_ second overtone of CH stretch, 1704–1780 nm for CH_3_ and CH_2_ first overtone of CH stretch, 2300–2370 nm for CH stretch in combination with CC stretch [36,37,38]. The absorption bands from 2100 to 2170 nm and absorptions around 1680 nm were contributed by CH stretch (–CH=CH–) and can be used to quantify the unsaturated fatty acids [39]. In general, the sample with high total fatty acid (TFA) contents possessed high absorption value in the wavelength range for CH stretch (Figure 1).

### 2.2. NIRS Models Based on C. vulgaris Data

Forty-five samples of *C. vulgaris* were randomly assigned to the calibration set, and the left 15 ones were assigned to the validation set. Calibration set was used to create NIRS model and validation set was to validate the model. The means, maximum values, minimum values and standard deviation of total fatty acids (TFA), palmitic acid (C16:0), stearic acid (C18:0), oleic acid (C18:1), linoleic acid (C18:2) and linolenic acid (C18:3) contents of 60 samples were determined by GC-MS and shown inTable 1. These five fatty acids are the common components of biodiesel [40]. In order to obtain a NIRS model suitable for predicting a fatty acid in unknown samples of *C. vulgaris*, the content range of the fatty acid in calibration and validation set should be as wide as possible [41]. To meet this need, the 60 samples were collected under different culture conditions and contained very wide concentration ranges of TFA, C16:0, C18:0, C18:1 and C18:3 (Table 1). Myristic acid (C14:0), palmitoleic acid (C16:1), hexadecadienoic acid (C16:2) and hexadecatrienoic acid (C16:3) were present in trace amounts (in total less than 5% of TFA in each sample) and thus not considered here.

**Table 1:** Descriptive statistics of the sample sets of *C. vulgaris* used for calibration and validation (Fatty acids expressed as mg/g dry cell weight).

Fatty Acid	Calibration Set (45 Samples)				Validation Set (15 Samples)			
	Mean	Max.	Min.	S.D.	Mean	Max.	Min.	S.D.
TFA	303.42	468.56	176.64	78.12	314.27	446.49	233.76	63.65
C16:0	59.29	114.55	29.00	23.41	62.33	105.28	40.78	19.61
C18:0	21.06	42.23	5.99	10.17	23.34	40.99	12.55	9.28
C18:1	67.81	112.97	29.90	21.39	71.68	104.22	47.59	17.54
C18:2	78.52	86.93	65.43	5.67	78.49	86.62	72.17	4.16
C18:3	63.62	108.11	33.20	19.40	66.24	100.15	47.63	15.15

Max.: maximum; Min.: minimum; S.D.: standard deviation.

The NIR spectra (wavelength range of 1000–2499 nm, WR I) and fatty acid contents data determined by GC-MS of *C. vulgaris* were combined by partial least squares 1 (PLS 1) regression with leave-one-out cross-validation. The resulting NIRS models for fatty acid quantification in *C. vulgaris* were named as CV-NIRS-WR I and shown in Table 2. The model (CV-NIRS-WR I) had a good performance for the prediction of TFA content, with root mean square error of calibration (RMSEC) (mg/g cell), multiple coefficient of determination (*R*^2^), root mean square error of cross validation (RMSECV) (mg/g cell), standard error of performance (SEP) (mg/g cell), the coefficient of determination (*r*^2^), and ratio of standard deviation of the validation set to standard error of prediction (*RPD*) being 5.81, 0.997, 7.15, 7.23, 0.994, and 10.83, respectively. The high *RPD* value suggested the feasibility of this model for broad applications, such as screening, quality control, and process control. As for the prediction of C16:0, C18:1 and C 18:3, the models had *RPD* values of over 6 and were therefore feasible for quality control use. When predicting C18:0, the *RPD* value of the model was 3.76 indicating possible screening use. In contrast, the model might be unsuitable for the prediction of C18:2, as the *RPD* values was less than 2. The poor prediction of CV-NIRS-WR I model for C18:2 may be attributed to the narrow range of C18:2 contents in *C. vulgaris* samples used for the model development (Table 1) [30]. It is well known that proteins (C–N and C=O bonds), polysaccharides (C–O bonds), and water (O–H bonds) have absorption at the wavelength range of 1880–2499 nm, which may interfere with the performance of NIR spectra for fatty acid analysis [42]. In order to minimize the interference caused by these compounds, we developed additional CV-NIRS models based on the data obtained from the wavelength ranges of 1030–1500 and 1600–1880 nm (WR II), where fatty acids show dominant absorbance over others. The model had an excellent performance for the prediction of TFA content, with RMSEC (mg/g cell), *R*^2^, RMSECV (mg/g cell), SEP (mg/g cell), *r*^2^, and *RPD* being 4.41, 0.998, 5.28, 6.47, 0.997, and 14.68, respectively. Besides, the RPD values of CV-NIRS-WR II model were higher than those of CV-NIRS-WR I for the prediction of TFA and individual fatty acids (Table 2). In contrast to CV-NIRS-WR I, RMSEC, RMSECV and SEP of CV-NIRS-WR II models for most fatty acids contents decreased significantly, which signified that precision and accuracy of prediction increased. Therefore, CV-NIRS-WR II was more suitable for rapid fatty acid composition analysis in *C. vulgaris*.

**Table 2:** Partial least squares 1 (PLS 1) analysis results of *C. vulgaris* using CV-NIRS WR I and WRII ^a^ models.

Model	RMSEC (mg/g Cell)	*R*^2^ ^b^	RMSECV (mg/g Cell)	SEP (mg/g Cell)	*r*^2^ ^c^	*RPD*
**Models developed with WR I (CV-NIRS-WR I)**						
TFA	5.81	0.997	7.15	7.23	0.994	10.83
C16:0	2.44	0.994	3.31	3.35	0.985	7.02
C18:0	2.53	0.969	2.68	2.94	0.951	3.76
C18:1	2.56	0.993	3.38	2.47	0.989	6.27
C18:2	2.65	0.888	3.16	3.95	0.604	1.78
C18:3	2.56	0.992	3.13	3.44	0.976	6.15
**Models developed with WR II (CV-NIRS-WR II)**						
TFA	4.41	0.998	5.28	6.47	0.997	14.68
C16:0	1.68	0.997	2.19	1.95	0.995	10.59
C18:0	1.54	0.989	1.62	1.61	0.984	6.22
C18:1	2.85	0.991	2.90	2.36	0.991	7.31
C18:2	1.96	0.940	2.18	3.98	0.678	2.58
C18:3	1.59	0.997	2.01	2.68	0.984	9.50

^a^ CV-NIRS-WR I: the models based on the spectra of *C. vugaris* in the wavelength ranges of 1000–2500 nm; CV-NIRS-WR II: the models based on the spectra of *C. vugaris* in the wavelength ranges of 1030–1500 and 1600–1880 nm; ^b^ *R*^2^: multiple coefficient of determination of calibration models; and ^c^ *r*^2^: coefficient of determination of regression models tested with validation sets.

### 2.3. NIRS Models Suitable for Three Species of Chlorella Simultaneously

CV-NIRS-WR II model, however, showed poor performance when predicting fatty acid composition in *C. protothecoides* and *C. zofingiensis*. This may indicate that the model built based on samples from a single strain is not suitable for other algal strains. In this context, we built new models by adding extra NIR spectra from 30 samples of *C. zofingiensis* and 69 samples of *C. protothecoides*. Briefly, 119 samples were randomly assigned to the calibration set, and the remaining 40 samples were assigned to the validation set. The means, maximum values, minimum values and standard deviation of TFA, C16:0, C18:0, C18:1, C18:2 and C18:3 contents of 159 samples determined by GC-MS were shown in Table 3. Likewise, other fatty acids in trace amounts were not considered in the present investigation.

**Table 3:** Descriptive statistics of the sample sets of *C. vulgaris*, *C. zofingiensis* and *C. protothecoides* used for calibration and validation (contents of various fatty acids expressed as mg/g dry cell weight).

Fatty Acid	Calibration Set (119 Samples)				Validation Set (40 Samples)			
	Mean	Max.	Min.	S.D.	Mean	Max.	Min.	S.D.
TFA	289.35	468.56	95.08	93.92	297.14	463.58	93.00	90.86
C16:0	46.57	114.55	17.13	18.71	49.48	111.97	16.64	21.62
C18:0	17.96	41.39	1.03	8.45	19.46	42.23	0.93	9.74
C18:1	100.96	198.52	22.13	50.87	98.59	201.93	26.10	47.86
C18:2	63.00	120.39	24.94	21.28	65.33	118.96	24.37	21.09
C18:3	39.05	108.11	5.70	23.14	43.50	100.15	5.99	25.50

Max.: maximum; Min.: minimum; S.D.: standard deviation.

Based on the spectra of WR II (wavelength range of 1030–1500 and 1600–1880 nm) and fatty acid composition measured by GC-MS of all 159 samples, a series of new NIRS models, namely, CVPZ-NIRS-WR II, were created. Calibration and validation performances were calculated and shown in Table 4. Among these models, the one for prediction of TFA content had the best performance, with RMSEC (mg/g cell), *R*^2^, RMSECV (mg/g cell), SEP (mg/g cell), *r*^2^, and *RPD* being 14.68, 0.988, 18.81, 24.16, 0.964, and 4.98, respectively. Although CVPZ-NIRS-WR II models had lower *RPD* values and higher RMSEC, RMSECV and SEP than CV-NIRS-WR II models for predicting C16:0, C18:0, C18:1 and C18:3 contents (Table 4), they demonstrated suitability to predict fatty acids in the three *Chlorella* species with the same NIRS models for the possible screening purpose. The NIRS models for fatty acids composition prediction in *C. protothecoides* and *C. zofingiensis* have been developed based on these 69 samples of *C. protothecoides* and 30 samples of *C. zofingiensis*, respectively. The model from *C. protothecoides* for prediction of TFA (C16:0 and C18:1) content had good performance with *R*^2^ and *RPD* being 0.992 (0.985 and 0.979) and 7.45 (4.56 and 2.80), respectively. As for the model from *C. zofingiensis*, they were, respectively, 0.998 (0.997 and 0.989) and 9.58 (9.81 and 5.61). Although the models based on 3 *Chlorella* species are not as good as those based on individual species, they are feasible for mutant screening use.

**Table 4:** PLS 1 analysis result for CVPZ-NIRS-WR II models.

Model	RMSEC (mg/g Cell)	*R*^2^ ^a^	RMSECV (mg/g Cell)	SEP (mg/g Cell)	*r*^2^ ^b^	*RPD*
TFA	14.68	0.988	18.81	24.16	0.964	4.98
C16:0	4.41	0.968	5.21	5.77	0.964	3.58
C18:0	2.36	0.961	2.56	2.87	0.957	3.29
C18:1	8.37	0.986	10.55	11.56	0.970	4.81
C18:2	6.78	0.949	7.25	7.67	0.932	2.92
C18:3	4.27	0.981	4.80	5.49	0.977	4.80

^a^ *R*^2^: multiple coefficient of determination of calibration models; ^b^ *r*^2^: coefficient of determination of regression models tested with validation sets.

## 3. Discussion

Near-infrared spectroscopy (NIRS) consists of complex overtones and combinations of molecular vibrations [29]. In contrast to sharp absorption peaks in the infrared region, there is no strong and unique band associated with a special chemical bond in the NIR spectrum [39]. However, the corrections can be established between information in the NIRS and measured values by using chemometric methods, such as PLS 1 regression [43,44]. With appropriate NIRS models developed, the compounds of unknown samples can be determined rapidly by their NIR spectra, including carbohydrate, protein, ash content and lipid [28,29,30,31,32,33].

Recently, NIRS has also been applied to the characterization of fatty acids in meat and food power samples [23,24,25,26,27]. But the models developed in these reports need improvements for better prediction of some fatty acids. For example, in the study of Fernandez-Cuesta *et al.* [24], only C18:1 and C18:2 showed good prediction, with *R*^2^ being 0.97 and 0.98, and *RPD* being 5.37 and 7.35, respectively; in contrast, the prediction performance for C16:0 (*R*^2^ = 0.54, *RPD* = 1.41) and C18:0 (*R*^2^ = 0.51, *RPD* = 1.44) was far less acceptable. Fatty acids varying in chain length and unsaturation level possess different near-infrared spectra [22]. Within the wavelength range of 1000–2499 nm, NIRS spectra also contain strong signals contributed by other compounds, including proteins (C–N and C=O bonds), polysaccharides (C–O bonds), and water (O–H bonds) [38,42]. In order to minimize the interference caused by these compounds, we developed CV-NIRS-WR II models by selecting the NIRS spectra within the wavelength ranges of 1030–1500 and 1600–1880 nm, where fatty acids show dominant absorbance. These models demonstrated excellent performances for predicting the contents of TFA, C16:0, C18:0, C18:1 and C18:3 in microalgae, with RMSECV, *R*^2^ and RPD being 1.62–5.28 mg/g cell, 0.991–0.998 and 7.31–14.68, respectively (Table 2), superior to the previous reports mentioned above.

Microalgal biodiesel has been considered as a promising alternative to fossil fuels, but challenges remain to be addressed to improve its production economics [1]. Efforts have been made to search an ideal algal strain as the biodiesel feedstock, which is expected to have not only fast growth rate and high lipid content but also great fatty acid composition, as the key properties of a biodiesel are largely determined by the composition of its fatty acid methyl esters (FAMEs) [4,45]. Currently, fatty acid profiles determination is mainly based on GC-FID/GC-MS, which is energy-intensive and time-consuming and thus less suitable for high-throughput screening purposes. Our work, for the first time, established a novel NIRS technique for rapid determination of fatty acids in microalgae. Unlike GC/GC-MS, the NIRS based fatty acid determination is free of cell disruption, oil extraction and transesterification, can be done in a few seconds, and has great potential in high-throughput applications of algae screening for better biodiesel production.

## 4. Experimental Section

### 4.1. Chlorella Species and Culture Conditions

Three *Chlorella* species of *Chlorella vulgaris* (Carolina 15-2075), *Chlorella protothecoides* (CSIROCS-41), and *Chlorella zofingiensis* (ATCC 30412) were grown heterotrophically in the medium with a series of nitrate concentration. The inocula were prepared by culturing the microalgae in 500-mL Erlenmeyer flask with 200 mL of Kuhl medium at 25 °C for 4 days with orbital shaking at 150 rpm in the dark. The seeds of *C. vulgaris*, *C. protothecoides* and *C. zofingiensis* were inoculated to 200 mL of fresh modified Basal medium, modified Basal medium and modified Kuhl medium, respectively, at a 5% (*v*/*v*) inoculums size for batch culture in 500-mL Erlenmeyer flask. The modified Basal medium contained (per liter) different concentration of KNO_3_ from 0.75 to 10 g, 40 g glucose, 1.25 g KH_2_PO_4_, 1 g MgSO_4_·7H_2_O, 0.5 g EDTA·Na_2_, 0.1142 g H_3_BO_3_, 0.111 g CaCl_2_·2H_2_O, 0.0498 g FeSO_4_·7H_2_O, 0.0882 g ZnSO_4_·7H_2_O, 0.0142 g MnCl_2_·4H_2_O, 0.0157 g CuSO_4_·5H_2_O, 0.0049 g Co(NO_3_)_2_·6H_2_O, and 0.0071 g MoO_3_. The modified Kuhl medium was consisted of (per liter) different concentrations of KNO_3_ from 0.75 to 10 g, 40 g glucose, 0.62 g NaH_2_PO_4_·H_2_O, 0.089 g Na_2_HPO_4_·2H_2_O, 0.247 g MgSO_4_·7H_2_O, 14.7 mg CaCl_2_·2H_2_O, 6.95 mg FeSO_4_·7H_2_O, 0.061 mg H_3_BO_3_, 0.169 mg MnSO_4_·H_2_O, 0.287 mg ZnSO_4_·7H_2_O, 0.0025 mg CuSO_4_·5H_2_O, and 0.01235 mg (NH_4_)_6_MO_7_O_24_·4H_2_O. The pH values of modified Basal medium and modified Kuhl medium were adjusted to 6.1 and 6.5, respectively, prior to autoclaving. After inoculating, flasks were incubated at 25 °C in an orbital shaker at 150 rpm in the dark for 10 days. All samples were harvested and lyophilized for the collection of NIR spectra and fatty acid analysis. In total, 30 samples of *C. zofingiensis*, 69 samples of *C. protothecoides* and 60 samples of *C. vulgaris*, which contained various lipids contents, were collected and lyophilized.

### 4.2. Fatty Acid Analysis

Twenty milligrams of lyophilized algal cells were incubated in a solvent mixture (1 mL toluene, 2 mL 1% sulfuric acid in methanol (*v*/*v*) and 0.8 mg heptadecanoic acid in 0.8 mL hexane as the internal standard) overnight at 50 °C for transesterification to form fatty acid methyl esters (FAMEs). FAMEs were then extracted three times with hexane in a reciprocating shaker. The FAMEs were analyzed by using a GC-MS-QP 2010 SE (Electron Ionization type) gas chromatograph-mass spectrometer (SHIMADZU, Kyoto, Japan) and a Stabilwax-DA capillary column (30 m × 0.25 mm × 0.25 μm) (SHIMADZU, Kyoto, Japan). Helium was used as the carrier gas. The injection temperature, ion temperature and interface temperature were set at 250, 200 and 260 °C, respectively. The initial column temperature was set at 150 °C. The column temperature subsequently rose to 200 °C at 10 °C/min and then to 220 °C at 6 °C/min, followed by a hold at 220 °C for 10 min. FAMEs were identified by NIST 11 mass spectral library (NIST/EPA/NIH mass spectral library, 2011 edition). The quantities of individual FAMEs were calculated by the peak areas according to the total ion chromatogram (TIC) using heptadecanoic acid as the internal standard.

### 4.3. NIR Spectra Collection

NIR spectra were collected by a Portable NIRS Analyzer (SupNIRS 1550, Focused Photonics Inc., Hangzhou, China). Temperature and relative humidity conditions during scanning ranged from 22 to 26 °C and from 35% to 45%, respectively. About 200 mg biomass of each sample was packed into a 1.5-mL Eppendorf tube for collecting NIRS. Diffusely reflected radiation was detected with optical fiber probe from 1000 to 2499 nm at a 1 nm resolution. The NIRS of each individual sample were obtained by averaging 5 parallel spectra.

### 4.4. Regression Model Development

Spectra data of all samples were converted and imported into the chemometrics software of the Unscrambler version 9.7 (CAMO, Trondheim, Norway). First, spectra were pretreated with the approach of Savitzky-Golay smoothing filter to preserve the features of distribution. Second, the first order derivatives were computed by the convolution (Savitzky-Golay) method to reduce peak overlap and eliminate baseline shift [46]. Then the algorithms of multiplicative scatter correction (MSC) were used for polynomial baseline correction to remove the multiplicative interference of scatter and particle size. The last preprocessing method was mean centering, which translated the collected data to the origin of the multivariate space where analysis would be performed. We developed NIRS models to predict the content of various fatty acids using PLS 1 regression with leave-one-out cross-validation. Every PLS 1 model was developed by calibration and validation set, which was composed of three quarters and one quarter of pretreated spectra, respectively.

### 4.5. Calibration Performance

In common, the calibration performance of each regression model was evaluated by RMSECV, RMSEC, and *R*^2^. The regression models were tested with the validation sets and three parameters, namely SEP, *r*^2^ and *RPD*, were calculated to assess the predictability. Notably, in the same concentration range, the accuracy of prediction result increases with the *RPD* value. *RPD* values of <2 indicate that the prediction result by the model is unacceptable; *RPD* values of 2–5 indicate the model is suitable for screening; *RPD* values of >5 indicate the model is suitable for quality control and even process control, and *RPD* values of >8 indicate the model is suitable for all possible applications [39]. Besides, the closer to 1 the *R*^2^ or *r*^2^ is, the more accurate the NIRS model is. As for RMSECV, RMSEC, and SEP, the smaller the better.

## 5. Conclusions

The key properties of biodiesel are largely determined by the fatty acid methyl ester profile. Therefore, when evaluating the feasibility of biodiesel feedstocks, the fatty acid composition should be considered as an important indicator. The present study developed a novel near-infrared spectroscopy (NIRS) technique for rapid and reliable analysis of fatty acids in microalgae, which can be done within a few seconds and requires only a small amount of samples. The optimized NIRS method demonstrated to have a good performance for the quantification of fatty acids across *Chlorella* species. In a word, compared to the traditional GC-mediated analyses, the reliable NIRS technique described here bypasses the involvement of cell disruption, oil extraction and transesterification and is thus easier to conduct and more environmentally friendly, and has great potential for screening purposes, in particular the high-throughput screening of oleaginous microalgal fatty acids for biodiesel uses.

## References

[1] Chisti Y. (2007). Biodiesel from microalgae. Biotechnol. Adv..

[2] Chisti Y. (2008). Biodiesel from microalgae beats bioethanol. Trends Biotechnol..

[3] Chisti Y., Yan J.Y. (2011). Energy from algae: Current status and future trends algal biofuels—A status report. Appl. Energy.

[4] Huang G.H., Chen F., Wei D., Zhang X.W., Chen G. (2010). Biodiesel production by microalgal biotechnology. Appl. Energy.

[5] Liu J., Huang J.C., Sun Z., Zhong Y.J., Jiang Y., Chen F. (2011). Differential lipid and fatty acid profiles of photoautotrophic and heterotrophic *Chlorella zofingiensis*: Assessment of algal oils for biodiesel production. Bioresour. Technol..

[6] Hu Q., Sommerfeld M., Jarvis E., Ghirardi M., Posewitz M., Seibert M., Darzins A. (2008). Microalgal triacylglycerols as feedstocks for biofuel production: Perspectives and advances. Plant J..

[7] Li J., Peng X., Luo M., Zhao C.J., Gu C.B., Zu Y.G., Fu Y.J. (2014). Biodiesel production from *Camptotheca acuminata* seed oil catalyzed by novel Bronsted-Lewis acidic ionic liquid. Appl. Energy.

[8] Liu J., Huang J.C., Fan K.W., Jiang Y., Zhong Y.J., Sun Z., Chen F. (2010). Production potential of *Chlorella zofingienesis* as a feedstock for biodiesel. Bioresour. Technol..

[9] Liu J., Huang J.C., Jiang Y., Chen F. (2012). Molasses-based growth and production of oil and astaxanthin by *Chlorella zofingiensis*. Bioresour. Technol..

[10] Sun Z., Zhou Z.G., Gerken H., Chen F., Liu J. (2014). Screening and characterization of oleaginous *Chlorella* strains and exploration of photoautotrophic *Chlorella protothecoides* for oil production. Bioresour. Technol..

[11] Prajapati S.K., Malik A., Vijay V.K. (2014). Comparative evaluation of biomass production and bioenergy generation potential of *Chlorella* spp. through anaerobic digestion. Appl. Energy.

[12] Ashraful A.M., Masjuki H.H., Kalam M.A., Rahman S.M.A., Habibullah M., Syazwan M. (2014). Study of the effect of storage time on the oxidation and thermal stability of various biodiesels and their blends. Energy Fuels.

[13] Knothe G. (2008). “Designer” biodiesel: Optimizing fatty ester composition to improve fuel properties. Energy Fuels.

[14] Park J.Y., Kim D.K., Lee J.P., Park S.C., Kim Y.J., Lee J.S. (2008). Blending effects of biodiesels on oxidation stability and low temperature flow properties. Bioresour. Technol..

[15] Ramírez-Verduzco L.F., Rodríguez-Rodríguez J.E., Jaramillo-Jacob A.D. (2012). Predicting cetane number, kinematic viscosity, density and higher heating value of biodiesel from its fatty acid methyl ester composition. Fuel.

[16] Tong D.M., Hu C.W., Jiang K.H., Li Y.S. (2011). Cetane number prediction of biodiesel from the composition of the fatty acid methyl esters. J. Am. Oil Chem. Soc..

[17] Nascimento I.A., Marques S.S.I., Cabanelas I.T.D., Pereira S.A., Druzian J.I., de Souza C.O., Vich D.V., de Carvalho G.C., Nascimento M.A. (2013). Screening microalgae strains for biodiesel production: Lipid productivity and estimation of fuel quality based on fatty acids profiles as selective criteria. Bioenergy Res..

[18] Wei L., Huang X., Huang Z., Zhou Z. (2013). Orthogonal test design for optimization of lipid accumulation and lipid property in *Nannochloropsis oculata* for biodiesel production. Bioresour. Technol..

[19] Andruleviciute V., Makareviciene V., Skorupskaite V., Gumbyte M. (2014). Biomass and oil content of *Chlorella* sp., *Haematococcus* sp., *Nannochloris* sp and *Scenedesmus* sp under mixotrophic growth conditions in the presence of technical glycerol. J. Appl. Phycol..

[20] Huang X.X., Wei L.K., Huang Z.Z., Yan J.Q. (2014). Effect of high ferric ion concentrations on total lipids and lipid characteristics of *Tetraselmis subcordiformis*, *Nannochloropsis oculata* and *Pavlova viridis*. J. Appl. Phycol..

[21] Holman R.T., Edmondson P.R. (1956). Near-infrared spectra of fatty acids and some related substances. Anal. Chem..

[22] Perez-Vich B., Velasco L., Fernandez-Martinez J.M. (1998). Determination of seed oil content and fatty acid composition in sunflower through the analysis of intact seeds, husked seeds, meal and oil by near-infrared reflectance spectroscopy. J. Am. Chem. Soc..

[23] Coppa M., Ferlay A., Leroux C., Jestin M., Chilliard Y., Martin B., Andueza D. (2010). Prediction of milk fatty acid composition by near infrared reflectance spectroscopy. Int. Dairy J..

[24] Fernández-Cuesta Á., Fernández-Martinez J.M., Company R.S.I., Velasco L. (2013). Near-infrared spectroscopy for analysis of oil content and fatty acid profile in almond flour. Eur. J. Lipid Sci. Technol..

[25] Guy F., Prache S., Thomas A., Bauchart D., Andueza D. (2011). Prediction of lamb meat fatty acid composition using near-infrared reflectance spectroscopy (NIRS). Food Chem..

[26] Riovanto R., de Marchi M., Cassandro M., Penasa M. (2012). Use of near infrared transmittance spectroscopy to predict fatty acid composition of chicken meat. Food Chem..

[27] Zamora-Rojas E., Garrido-Varo A., de Pedro-Sanz E., Guerrero-Ginel J.E., Pérez-Marín D. (2013). Prediction of fatty acids content in pig adipose tissue by near infrared spectroscopy: At-line *versus* in-situ analysis. Meat Sci..

[28] Brown M.R., Frampton D.M.F., Dunstan G.A., Blackburn S.I. (2014). Assessing near-infrared reflectance spectroscopy for the rapid detection of lipid and biomass in microalgae cultures. J. Appl. Phycol..

[29] Challagulla V., Walsh K.B., Subedi P. (2014). Biomass and total lipid content assessment of microalgal cultures using near and short wave infrared spectroscopy. Bioenergy Res..

[30] Laurens L.M.L., Wolfrum E.J. (2013). High-throughput quantitative biochemical characterization of algal biomass by NIR spectroscopy; multiple linear regression and multivariate linear regression analysis. J. Agric. Food Chem..

[31] Mayers J.J., Flynn K.J., Shields R.J. (2013). Rapid determination of bulk microalgal biochemical composition by Fourier-Transform Infrared spectroscopy. Bioresour. Technol..

[32] Mulbry W., Reeves J., Liu Y., Ruan Z.H., Liao W. (2012). Near- and mid-infrared spectroscopic determination of algal composition. J. Appl. Phycol..

[33] Wagner H., Liu Z.X., Langner U., Stehfest K., Wilhelm C. (2010). The use of FTIR spectroscopy to assess quantitative changes in the biochemical composition of microalgae. J. Biophotonics.

[34] Gardner R., Peters P., Peyton B., Cooksey K.E. (2011). Medium pH and nitrate concentration effects on accumulation of triacylglycerol in two members of the chlorophyta. J. Appl. Phycol..

[35] Praveenkumar R., Shameera K., Mahalakshmi G., Akbarsha M.A., Thajuddin N. (2012). Influence of nutrient deprivations on lipid accumulation in a dominant indigenous microalga *Chlorella* sp., BUM11008: Evaluation for biodiesel production. Biomass Bioenergy.

[36] Plans M., Simo J., Casanas F., Sabate J. (2012). Near-infrared spectroscopy analysis of seed coats of common beans (*Phaseolus vulgaris* L.): A potential tool for breeding and quality evaluation. J. Agric. Food Chem..

[37] Prieto N., Dugan M.E.R., López-Campos Ó, Aalhus J.L., Uttaro B. (2013). At line prediction of PUFA and biohydrogenation intermediates in perirenal and subcutaneous fat from cattle fed sunflower or flaxseed by near infrared spectroscopy. Meat Sci..

[38] Workman J. (2001). Handbook of Organic Compounds.

[39] Kim Y., Kays S.E. (2009). Near-infrared (NIR) prediction of *trans*-fatty acids in ground cereal foods. J. Agric. Food Chem..

[40] Pinzi S., Rounce P., Herreros J.M., Tsolakis A., Dorado M.P. (2013). The effect of biodiesel fatty acid composition on combustion and diesel engine exhaust emissions. Fuel.

[41] Laurens L.M.L., Wolfrum E.J. (2011). Feasibility of spectroscopic characterization of algal lipids: Chemometric correlation of NIR and FTIR spectra with exogenous lipids in algal biomass. Bioenergy Res..

[42] Workman J.J., Weyer L. (2012). Practical Guide and Spectral Atlas for Interpretive Near-Infrared Spectroscopy.

[43] Bao Y.D., Kong W.W., Liu F., Qiu Z.J., He Y. (2012). Detection of glutamic acid in oilseed rape leaves using near infrared spectroscopy and the least squares-support vector machine. Int. J. Mol. Sci..

[44] Jiang B., Huang Y.D. (2007). Near infrared spectroscopy for on-line monitoring of alkali-free cloth/phenolic resin prepreg during manufacture. Int. J. Mol. Sci..

[45] Ordog V., Stirk W.A., Balint P., Lovasz C., Pulz O., van Staden J. (2013). Lipid productivity and fatty acid composition in *Chlorella* and *Scenepdesmus* strains grown in nitrogen-stressed conditions. J. Appl. Phycol..

[46] Davies A.M.C., Grant A. (1987). Near-infrared analysis of food. Int. J. Food Sci. Technol..

